# Gynæcology in Japan

**Published:** 1877-09

**Authors:** A. Wernich

**Affiliations:** Yeddo


### Translations.

### GYNÆCOLOGY IN JAPAN.

By Dr. A. Wernich, of Yeddo.

[TRANSLATED BY DR. JAMES I. TUCKER, CHICAGO.]

(Continued.)

### GYNÆCOLOGICAL DISEASES. I. OVARIES.

In examining the materials of ovarian disease, which my colleague, Dr. Schultze, has collected, and which he kindly permitted me to use, I find no account whatever of Oöphoritis. This is not to be attributed altogether to the caution which is universally observed in the diagnosis of these extreme puerperal conditions, but to the fact that of all the cases not even one has awakened the suspicion that this condition existed.

Encysted and indurated tumors were, on the contrary, met with in 21 well-marked and rather advanced cases. These cases were shared by Dr. Schultze and myself. He had nine and I had twelve, since those patients who came to the hospital for the purpose of operations were immediately assigned to the surgical department. Among my twelve cases were four clearly cystic, fluctuating tumors, one of which proved upon puncture to be a multilocular cystoid. Eight of my cases were surrounded with solid matter, among which were two very hard, apparently perfectly solid tumors. I am of the opinion that we must look upon this preponderance of the latter as accidental, especially as Dr. Schultze out of nine cases found three (including those recorded somewhat in detail below), as solid tumors (cysto-sarcoma or teratoid).

I omit here intentionally the anatomical description of the preparations derived from ovariotomy, because Dr. Schultze has reserved for himself the report of operations, and the description of tumors must necessarily form an important part of this report.

I give instead, the result of clinical observation, as follows;

The growth of tumors, of the encysted as well as the indurated character, was, on the whole, slow. Though the Japanese women are attentive to *Katameri* (literally translated; lumpy masses), in the abdomen, yet only one could give an account of a tumor whose growth was really rapid. Three or four years was the interval after the discovery of the usual tumor, as large as the fist, to the time when it had become troublesome through size and weight. After Dr. Shultze had operated on three cases of ovarian tumor, in quick succession, a number of new cases made their appearance. Yet in private practice I met with a number of ovarian tumors which could only be felt, but which had existed five to six years.

Relief by spontaneous evacuation from the outside, or through the peritoneum, is apparently not at all known. Puncture is regarded necessary by physicians and the women, and has been a means of palliation for a long time. Prior to the first ovariotomy in Japan by Dr. Schultze, with my assistance, March 25, 1875, ovarian tumor was supposed to be a fatal disease.

I have never observed any remarkable alteration in, or sudden disappearance of these tumors. Inflammation in and about the tumor was noticed in one case.

Functional disturbances of other organs were, it is true, distinctly observed, but not felt, in excessive degree. In my twelve cases ascites was proven to exist in six, by physical examination, and in three by puncture and examination of the fluid contents. Albuminuria, cachexia, and marked disturbances of the stomach and alimentary canal were rare, on the contrary there was always considerable dyspnoea.

According to age the cases stand as follows :

| | | |
|---|---|---|
| At 11 years of age, | - - - - | 1 case. |
| From 20–30 years of age, | - - - - | 4 cases. |
| " 30–40 " " " | - - - | 9 " |
| " 40–50 " " " | - - - - | 5 " |
| " 50–60 " " " | - - - | 2 " |
| At 62 years of age, | - - - - | 1 case. |

It was impossible to discover any single aetiological influence, neither heredity, early or late appearance of menstruation, earlier or later marriage, frequent confinments, nor complication with other gynæcological diseases.

In my twelve cases, there was sudden suppression of menstruation in two; the period was irregular in nine; it continued perfectly regular in one.

Pains in the abdomen, generally referred to one side, was the first symptom in ten cases. Suppression of the urine or dysuria was observed only in two cases. Severe constipation occurred six times. Only once was the tumor unexpectedly discovered.

Neither Dr. Schultze nor I can contribute anything to lessen the difficulties in diagnosis, as the cases, as a whole, had been for a long time developed, and their true character easily distinguishable. Once, in a very respectable old Japanese woman, who, at my first visit, objected to an inward examination, I was uncertain whether I had to deal with ascites alone, or cysts complicated with ascites. The trocar enabled me to decide that the latter opinion was correct.

The following case, which Dr. Schultze kindly gave me for this communication, deserves especial mention.

Iwata. *Child* 11 *years old.*

Previously well. In January, 1875, she noticed, herself, in the left side of the abdomen a swelling about the size of a hen's egg, movable and destitute of sensation. Despite the remedies used, the swelling increased more and more. Since May, 1875, the patient has been obliged to go to stool several times a day. The discharge was not diarrhœtic, but somewhat softer than usual. After each passage, she experienced some pain in the region of the stomach; the emptying of the bladder, also, must be attended to oftener than formerly. For 20 days, feeling of weight and numbness in right foot, with some ataxy.

*Status praesens* from November 24, 1875. A maiden of healthy appearance, developed in proportion to her age. Subcutaneous connective tissue moderately adipose, mucous membrane somewhat pale. The belly is remarkable, being swelled up in a very irregular manner. There are especially two bunches, one in the regio mesogastrica dextra, the other in the regio hypogastrica media. The circumference of the abdomen, on a level with the umbilicus, is 58 Cm., on a level with the spinæ ant. sup., 62.5 Cm. Upon palpation, we feel a tumor in the abdominal cavity, filling the lower part of the cavity completely, and extending to the right as far as the lower lobe of the liver. From the lower part of the abdomen there extended a cord-like prolongation into the pelvis. The swelling had a knotty hardness, soft and elastic in the prolongation mentioned. The percussion impulse is absolutely dull over the tumor. The tumor is movable, and seems to be attached in the pelvis only. No tumor is discoverable upon rectal examination.

Exploration per vaginam was unavailable, on account of the natural obstacles presented by the genital organs of a child.

All the other organs were sound; the general condition, with the exception of difficulty of defecation and urination, intact. The patient was examined in the poli-clinic; she came several weeks, without change.

Diagnosis: Tumor ovarii dextri-teratoides (?)

### II. UTERUS.

### A. INFLAMMATORY AFFECTIONS.

I have never realized before so fully and unequivocally the quick and strong participation of the genital system in the affections of the whole female organism. While the habits which the Japanese women observe at the time of their menstruation, their slowness and cautiousness of bodily movements, the remarkably quiet temperament of the women and girls, which has already been referred to as peculiar to them, protect them almost absolutely from many very common causes and frequent occurrences of various uterine diseases, nevertheless scanty and interrupted mensturation occurs almost instantly as the consequence of severe internal disorder. I have endeavored to avoid confounding here cause and effect mistaking the deleterious consequences of an endometritis or chronic infarction with the vaguely distinguishable forms of disease which proceed from other internal organs. When I consult my notes and observe how seldom *trauma*, colds, and puerperal disorders figure among the forerunners of uterine affections, and how often, on the other hand, dysentery, a typhus, a yearly-recurring bronchial catarrh, and one or several attacks of hæmoptysis were clearly related to the existing trouble, thereby I am involuntarily impressed with the thought, by no means new but not entertained by all gynæcologists, that we should not only form a correct judgement concerning the local genital condition, but examine every gynæcological case in all its mainfold relations. It is to be hoped that the remark of a colleague, now dead, may not be general, a remark which he made with a sort of grim pleasure, to the effect that "he did not recognize women as old patients till he had them on the examination chair, and saw them *from below.*"

Those conditions which bore most frequently ætiological relations to *chronic hypertrophy of the cervix*, (pardon me for classifying this under "inflammatory affections") were the gradually developing chronic diseases of the lungs, especially infiltration with progressive tuberculization occurring after hæmoptoë, and cardiac dilitation, according to Beriberi. Next should be mentioned as by no means uncommon typhus, chronic gastric catarrh during the hot months and simple bronchial catarrh. It is not necessary to say that subinvolution in puerperium did not fail to appear, and also anæmia and hydræmia congenital, or acquired in childhood.

The form was generally that of a very widely expanded, swollen (ectropioned) vulva, some elongation of the whole organ, and prominent, dilated and over-filled blood-vessels. Erosions were observed in more than half the cases, deep lesions of the mucous membrane never. Good results followed the therapeutic means employed, which consisted of occasional heroic section of the dilated vessels, glycerine tampons, reclined position in bed, and mild astringent injections of gradually diminished temperature. Erosions healed much more promptly under the use of solutions of carbolate of zinc, or of carbolized oil, than of argentum nitricum, or under the use of some of the stronger astringents. One constitutional peculiarity of the Japanese women worked admirably against the danger from all acute inflammatory conditions of the uterus, namely, a very sluggish reaction of the whole mucous membrane. Puerperal endometritiden of considerable intensity, and apparently of a character not devoid of danger, got well quickly and surely under moderate mercurialization, and regular syringing with carbolic and salicylic acids.

B. MORBID GROWTHS.

If only one carcinoma uteri came to hand among the only 13 gynæcological patients, who, as out-patients, were immediately transferred to the surgical department, it should not be looked upon as anything particularly unusual. If, however, among the other 74 cases consigned to me and called "gynæcological," not a single carcinoma of the cervix appeared, the remark will appear justifiable that in Japan, at least in these parts of the country (from which the reputation of European physicians is continually drawing patients to the capital), carcinoma of the uterus is a very rare disease. In confirmation of this, my assistants were quite astonished, upon hearing me describe uterine cancer as a frequent cause of death, and several of them otherwise well-educated men, knew nothing of the disease in question even from hearsay. In respect to the ætiological significance of cancer, to which in many new and good works on the subject of gynæcology more and more space is being given, I cannot avoid referring once more to my general characteristics of the Japanese women. If, indeed, it is true that the influence of continual emotional excitement is to be regarded as a cause, we can almost understand how the women of a nation who live on so quietly, cheerfully, and without powerful emotion, as is the case with the Japanese women, how such women, I say, may be afflicted with cancer with remarkable infrequency. The inquiring pathologist who assigns reverses of fortune, etc., as the cause of so and so much per cent. of uterine cancer, only needs to take a short step into the domain of morals, to meet the most terrible admonition, and how near to my observation (surely not altogether accidental) that carcinoma of the uterus is very rare, is the conclusion that: *uterine cancer in the overwhelming majority of cases, is a consequence of the social and conjugal misery of our great cities!*

The above-mentioned single case, which occurred in a woman 46 years old, had already *verjauchung* (foul discharge), and perforation of the vagina, and the patient was much emaciated. She died after a two-weeks' sojourn in the surgical department.

*Sarcoma of the fundus uteri* I have witnessed in one patient, whom I was called to see in consultation with a Japanese physician, a few days before her death. There were large pieces which were microscopically distinguished as sarcoma, and in the fundus soft, friable irregular masses of the tumor, a part of which could easily be reached and removed by the finger. Yet the strength of the patient had so far failed, that for the removal of the fundus it would have required a certain improvement in the general condition, which was now impossible.

As a very common affection here I must speak of fibroma and fibro-myoma of the uterus. There were nine cases, of which four occurred in private practice, and five in the clinic. Of the latter, two left when the troublesome pains were relieved and the profuse bleeding had stopped, which was promptly after the use of ergotin injections. They left because these were painful to them. One left in eight and the other in eleven days. The other three cases deserve a short special mention.

Case I. (Dr. Schultze). Ito, 28 years old. Since September, 1875, pains in abdomen and irregular hemorrhages. In January, 1876, the bleeding persisted, though in moderate degree, twenty-five days. She had never been pregnant.

February 28th, 1876, the portio vaginalis completely anteverted. In the posterior concavity of the vagina an irregular, knotty, hard tumor. This can be distinctly felt from the rectum. It almost completely fills the rectum in the upper part, and extends so far upward, that its superior surface cannot be reached. The tumor is plainly attached to the posterior wall of the uterus. It cannot be felt distinctly in the abdomen. Uterine sound can be introduced but three cm., speculum examination reveals nothing of importance.

Case II. (My own observation.) Nurse, 48 years old. She complains of sense of weight and afterward bearing down sensations in the abdomen which have lasted two years. In September, 1875, felt, while at stool, a hard, labor-like pressure, and discovered, upon feeling in the vagina, a smooth, firm and soft bleeding body. It was two months before, on account of repeated hemorrhages which reduced her strength to a great degree, she concluded to take to her bed in the medical ward, and the following was discovered upon examination.

Just behind the lesser lips there was found a tumor of the size of a man's fist, of somewhat unequal, firm, and elastic consistency, closely encompassed every where by the vaginal walls. The mucous membrane of the vagina was smooth and very vascular. It was only possible to feel a fleshy protuberance (the anterior lips of the os uteri) after laboriously pressing up two fingers. In front the tumor obstructed the admission of the fingers, yet the character of the swelling is made out with sufficient clearness by sound and rectal examination, as one proceeding out of the uterus. The patient suffered considerably from hemorrhage and pain, and was very much reduced. We would under these circumstances have decided to remove the fibromyomal growth, had we been able to reach a pedicle. Twice daily injections of ergotin, to which this patient willingly submitted. The pains ceased first, then long cessation of the bleeding, which up to that time was almost continual. Gradually, as a consequence of the perpetual contraction of the uterus induced by the ergotin, a distinct pedicle began to form, up as far as (now after two and a half months) we are able to feel even to the peripherally-perfect circumference through the mouth of the womb. Yet the patient at this time felt so well as the result of internal and external use of liq. ferri sesquichloridi (she had not lost a drop of blood for fifty days), that an operation did not seem to be indicated. The tumor has become much firmer and somewhat reduced in size.*

Case III. (My own observation.) Madzusan, 34 years of age.

On the 24th of August, 1875, there was presented to me under the false diagnosis of "procidentia uteri" a patient with fibroid almost as big as as a child's head. The tumor, which for 13 days had protruded from the uterus and the vulva, was almost ulcerated off on its entire upper surface, and was covered with grayish brown, masses. It was attached to the somewhat inverted anterior uterine wall, by a thick pedicle of the thickness of two thumbs, and emitted a pestiferous stench. The patient could scarcely be said to be alive, she had lost so much blood. Over the symphysis and per rectum we felt a hard mass, apparently attached to the uterine wall, but it afterwards proved to be a flat intramural fibroid of the posterior wall. I concluded, under necessary precautions, to take off the projecting portion of the tumor by means of the galvano-cautery. The examination of the tumor revealed a simple fibrous structure; the ulceration of the upper surface was entirely conditioned by the frequent change of position of the patient, lying now upon her back and again sitting up, on account of the discomfort occasioned by the heavy hanging tumor, and conditioned also by the injurious effects of excessive heat.

The patient was perfectly well in three days. The cut end of the pedicle, which withdrew more and more into the uterine cavity, was entirely healed in eight days. The efforts to still further reduce the size of the part of the fibrous growth which was situated in the walls of the uterus, was only partially successful.

---

* The operation was, however, performed in the middle of June by means of the Ecraseur.

*C. Alterations in form and position.*

Twenty-three cases came under my observation, wherein there were changes in the position of the uterus from different causes; three of them were treated by pessaries, and eleven with other uterine supports. In some resorbents upon tampons, or in the form of suppositories were used; in others the patients were caused to lie constantly on the belly, and treatment of circumscribed parts of the vagina resorted to.

*Hæmatometra* has come only once to my notice. It exhibited a simple imperforatio hymenis and retention of the menstrual fluid, of twenty months standing. The case was cured by a simple crucial incision, and avoidance of pressure during the discharge.

*Absence of the uterus*, with a very short vagina (2½ cm.) was observed in one person twenty-one years old, who was otherwise very well developed. Upon synchronous palpation per rectum and vagina, we could feel above the cul-de-sac of the vagina a movable, smooth body, of the size of an almond.

*Atrophy of the uterus* seemed very frequently to appear very soon after the menopause. At least I have found it in all old women from 50 to 60 years of age, whom I examined in part for the sake ot being thorough, and of the students. We found a remarkably small, shrivelled and bloodless portio vaginalis hanging down in the vagina like a fold of skin, and upon examination with the sound, a uterine cavity of only 3 to 4 cm.

III. AFFECTIONS IN THE NEIGHBORHOOD OF THE UTERUS.

What I have already mentioned while speaking of puerperal endometritiden and metritiden concerning a favorable, often almost reactionless course, may be reiterated in regard to peri and parametritic processes, and is true of the majority of cases. At least the older ones, and the practitioners among my assistant physicians, have had no experience with the severer results of these inflammations. The presumption that in parametritic inflammations of a suppurative character, and leading to rupture, the mild course may be transformed into one enormously sluggish, was corroborated by the case of a 27-year-old primipara, who, exactly 10 months after the inception of a fluctuating tumor, was relieved of the pus it contained. A few days after the confinement she felt pain in the left side, which was succeeded by a feeling of heavy pressure and the discovery of a swelling which gradually became softer and softer. The diagnosis was difficult, on account of the extraordinarily slow development of the fluctuation, the absence of reaction, and because the discharge was so long delayed. At last the exudation worked itself to the left side, and showed here, immediately over Poupart's ligament, with fever-symptoms, but without particular pain, a very soft fluctuating place of the size of a walnut, from which (after incision) there escaped not only in the left side the newly-accumulated, but on the right side the long pent-up matter. After the evacuation the patient recovered in a few days.

*Hœmatocele* seems to be very rare. To the native physicians it was wholly unknown. I myself saw it once in residuum after the resorption was quite far advanced. There is perhaps another way to explain it, viz., by the fact that the Japanese women are seldom on the street during the period of menstruation.

IV. DISEASES WHICH INVOLVE THE VAGINA AND THE EXTERNAL GENITAL ORGANS.

Excepting elephantiasis, these have been seen neither by Dr. Schultze nor myself. Women (and also men) with a particularly high grade of elephantiasis of the lower extremities and of the sexual organs, exhibited themselves sometimes in the booths in the neighborhood of the Temple as objects of charity. A case of condylomata lata and acuminata, which came under treatment in the surgical clinic was interesting to us on account of the monstrous collection of the characteristic eminences.

Connected with the notice of the remarkably prominent meatus of the urethra, I may mention three cases as pathological, in which the entire urethral protuberance had a sausage-like feel, with a very significant degree of prolapse of the vaginal parietes without anything more than a slight anteversion of the uterus. The patients stated that it was gradually superincumbent upon an incontinent nocturnal urination. All three were unmarried and sterile. The suffering was relieved by leeches, large glycerin tampons, and cold tannin injections repeated every half hour. In close relation to the urethral abnormalities stands the frequent development of urethral carunculæ, a condition of things which has thrice come under my observation. The severe burning pains and occasional hemorrhages were always relieved by a simple operation. In one case it was necessary to treat the carunclæ of the urethra not only by dilatation, but subsequently by cauterization.

Vagino-blennorrhœa were very stubborn and unyielding when they were sequels of inflammation. Quantities of muco-purulent matter welled up in the morning in spite of efforts to keep clean, and in spite of the application of pulverized and dissolved astringent to the flabby, unevenly-swollen, and ecchymosed mucous membrane of the vagina. For this condition, diluted liq. ferri sesquichlor. alternated with strong solutions of carbolic acid, were used.

